# Amoxicillin and metronidazole resistance of bacteria isolated from dental implants with peri-implant diseases: a pilot cross-sectional study

**DOI:** 10.1099/acmi.0.000946.v3

**Published:** 2026-02-11

**Authors:** Ismael Secundino, Yosahandy Palacios-Castañon, Nailea Zambrano-Pérez, Mayemi Pamela Santiago-Martínez, María Teresa Zermeño-Loredo, Juana Elizabeth Reyes-Martínez, Victor Nizet

**Affiliations:** 1 Facultad de Odontología, Universidad La Salle Bajío, León-Guanajuato, México; 2 Facultad de Veterinaria, Universidad La Salle Bajío, León-Guanajuato, México; 3 Private Practice, Querétaro, Querétaro, México; 4 Posgrado en Prostodoncia e Implantología, Facultad de Odontología, Universidad La Salle Bajío, León-Guanajuato, México; 5 Departamento de Biología, División de Ciencias Naturales y Exactas, Universidad de Guanajuato, Guanajuato, México; 6 Division of Host-Microbe Systems & Therapeutics, Department of Pediatrics, University of California San Diego, La Jolla, California, USA

**Keywords:** antibiotic resistance, bacterial, dental implants, peri-implantitis

## Abstract

Peri-implant mucositis is a reversible inflammatory lesion of the mucosa surrounding a dental implant, caused by the accumulation of bacterial plaque and biofilm formation, without bone loss. If peri-implant mucositis is not addressed, it can progress to peri-implantitis, characterized by significant inflammation and infection of the peri-implant mucosa accompanied by the loss of supporting bone. Clinical evidence suggests that the management of peri-implant infections consists of mechanical debridement of the implant, surgical intervention and the administration of antibiotics. However, limited information is available regarding antibiotic resistance in bacteria causing peri-implant diseases. This study is focused on assessing the antibiotic resistance of bacteria isolated from explanted dental implants with peri-implant infections to amoxicillin, clindamycin and metronidazole. To this end, biofilms were recovered using titanium curettes from dental implants of 10 patients with peri-implant infections: patients with peri-implant mucositis (*n*=4) exhibited redness, swelling or bleeding and absence of bone loss; patients with peri-implantitis (*n*=6) were diagnosed based on probing depth ≥6 mm and presence of bone loss. Antibiotic sensitivity was assessed using the Kirby–Bauer disc diffusion method in accordance with the Clinical and Laboratory Standards Institute at 10 µg per disc of amoxicillin, 30 µg per disc of clindamycin and metronidazole at a concentration of 50 µg per disc. The results were expressed as the diameters of inhibition zones for each antibiotic. Two peri-implant bacteria were identified by sequencing of their 16S rRNA. Peri-implant bacteria showed resistance to amoxicillin and metronidazole at 100% (10 out of 10). All isolates from dental implants with peri-implant infections (10 out of 10) were sensitive to clindamycin. Two isolates, M29 and P30 strains, were identified as *Streptococcus salivarius* by 16S rRNA sequencing. Our findings reveal emerging resistance to amoxicillin and metronidazole in clinical isolates from implants with peri-implant infections, yet bacterial susceptibility to clindamycin remains.

## Data Summary

The authors confirm that the data supporting the findings of this study are available within the article and supplemental materials.

## Introduction

Peri-implant diseases are classified into peri-implant mucositis and peri-implantitis. Peri-implant mucositis is a reversible inflammatory condition of the gingiva surrounding an osseointegrated dental implant [1]. In contrast, peri-implantitis represents a chronic inflammatory response characterized by the absence of supporting alveolar bone [2]. The reported incidence rates are 29.48% for peri-implant mucositis and 9.25% for peri-implantitis [3]. Risk indicators for peri-implant diseases include periodontitis, smoking, poor oral hygiene, accumulation of dental plaque, certain implant designs, residual cement and absence of keratinized mucosa [2,4,6]. Clinically, peri-implant mucositis presents as visible signs of inflammation, such as localized erythema, swelling and suppuration, along with bleeding on probing (BOP), but without bone loss [7]. Treating peri-implant mucositis involves eliminating dental biofilm accumulation on the implant. Failure to address this infection can lead to increased biofilm formation, an intensified inflammatory response and deeper peri-implant pockets, resulting in an increased probing depth (PD) to ≥6 mm, BOP and bone loss around the implant, a condition known as peri-implantitis [2,7]. Indeed, in periodontitis, the imbalance caused by overproduction of free radicals and diminished production of antioxidants induces a sustained inflammatory response [8]. Such oxidative stress is caused by mitochondrial dysfunction, impaired autophagy and increased pro-inflammatory cytokine release [8]. In 30 patients with periodontitis, miRNA-200b-3p and miRNA-200b-5p found in gingival crevicular fluid were associated with oxidative stress [9]. Both studies suggest that oxidative stress boosts inflammatory response in periodontitis [8,9].

The clinical treatment of peri-implant diseases focuses on eliminating bacterial biofilm from the implant, reducing inflammatory response and maintaining the stability of supporting bone. Interventions to treat peri-implant diseases include non-surgical approaches based on mechanical debridement and disinfection of the implant. If these treatments fail to prevent disease progression, surgical procedures are recommended, such as access flap incision, debridement of necrotic tissue, implantoplasty, bone grafting and the administration of systemic antibiotics [10]. Antibiotics are widely prescribed by dentists for oral infections. However, overprescribing and inappropriate administration of antibiotics are contributing to the emergence of antibiotic resistance in oral bacteria [11,13].

For instance, a systematic review by Ardila *et al.* [14] examined the antibiotic resistance in subgingival plaque samples of 214 dental implants from 168 patients with peri-implantitis. The review included four cross-sectional reports from 1995 to 2014 [14]. They found that micro-organisms showed a predominant resistance to amoxicillin, clindamycin, doxycycline, metronidazole and tetracycline [14]. A systematic review and meta-analysis by Roca-Millan *et al.* [15] compared 11 clinical trials published between 2008 and 2019 [15]. The study aimed to determine if the administration of antibiotics in patients after dental implant surgery, with at least 3 months follow-up period, could reduce implant loss. The antibiotics included amoxicillin or clindamycin [15]. The findings demonstrated a statistically significant reduction in early implant loss when antibiotics were administered. The implant failure rate in the antibiotic group was 1.55%, which was considerably lower than the 4.61% implant loss found in placebo groups [15]. Based on these results, antibiotic prophylaxis is recommended for dental implant interventions, as it significantly reduces the risk of early implant loss.

In dental practice, amoxicillin, clindamycin and metronidazole are routinely administered to treat peri-implant infections [16]. However, there is a need for an update of literature on antibiotic resistance in bacteria causing peri-implant infections. Thus, we evaluated the antibiotic resistance of bacteria isolated from explanted dental implants with peri-implant infections to amoxicillin, clindamycin and metronidazole. The identification of antimicrobial resistance in bacteria isolated from dental implants could benefit dental practice to treat dental infections with a specific antibiotic and limit the dissemination of antibiotic resistance to other oral bacteria.

## Methods

### Patient selection

A cross-sectional study on patients with peri-implant infections was conducted. Patients were recruited from February 2022 to February 2023 at the Department of Prosthodontics and Implantology of Universidad La Salle Bajio, Leon-Guanajuato, Mexico.

### Inclusion and exclusion criteria

For patient selection, the following inclusion criteria were defined: partially/fully edentulous patients with at least one failed dental implant, which required explanation; presence of peri-implant mucositis or peri-implantitis; titanium implants. The following exclusion criteria were used: patients who did not require explantation of implants; presence of non-titanium implants.

### Peri-implant disease diagnosis

Diagnosis of peri-implant mucositis was based on the presence of erythema, swelling and bleeding on probing (BOP) observed within 30 s, without bone loss [1]. Peri-implantitis was diagnosed by increased signs of inflammation, probing depth (PD) of ≥6 mm and loss of supporting bone by x-ray [2]. Aforementioned criteria were established by the European Federation of Periodontology and the American Academy of Periodontology at the 2017 World Workshop [1,2].

### Explantation of dental implants

Local anaesthesia of 4% articaine with 1:100,000 epinephrine was used before explantation of dental implants. The counter-torque ratchet technique was used for explanation of implants with intact connection and absence of implant fracture; the implant extraction screwdriver was screwed into the implant, and removal of the implant was done slowly in a counterclockwise direction [17]. To reduce pain and prevent infection, after explantation of dental implants, the administration of ibuprofen at a dose of 600 mg orally every 8 h and amoxicillin plus clavulanic acid (875/125 mg) every 12 h for 7 days was used.

### Collection of extracted dental implants and peri-implant bacteria cultivation

Immediately, all implants were disinfected with 0.2% chlorhexidine gel for 1 min. Because of the recent COVID pandemic, to avoid cross-contamination of implants with severe acute respiratory syndrome coronavirus 2 (SARS-CoV-2), 0.5% povidone-iodine was applied to all dental implants to inactivate virus [18]. Then, subgingival biofilms were recovered from the apex of screw-thread implants using titanium curettes (HuFriedy). Immediately, samples were cultured in Todd-Hewitt broth under anaerobic conditions at 37 °C. Then, bacteria were stored at −20 °C using 15% glycerol until analysis.

### Antimicrobial susceptibility testing

The disc diffusion susceptibility assay was carried out according to the procedure defined by the Clinical Laboratory Standards Institute (CLSI), as reported elsewhere [19]. The concentrations of amoxicillin, clindamycin and metronidazole were used based on previous reports [20,22]. Paper discs with amoxicillin (10 µg), clindamycin (30 µg) and metronidazole (50 µg) were applied on plates previously inoculated with peri-implant bacteria. Upon incubation under anaerobic conditions at 37 °C for 18 h, the diameters of inhibition zones were evaluated. Antibiograms were repeated five times with similar results. *Escherichia coli* ATCC 25922 was used as a negative control strain.

### Molecular analysis

#### DNA isolation and 16S rRNA sequencing

Genomic DNA (gDNA) was isolated as reported in the protocol by Aljanabi and Martinez [23] with brief modifications. Overnight liquid culture of 3 ml was centrifuged, the supernatant was eliminated and 200 µl of resuspension buffer was added (0.05 M Tris/HCl, pH=8; 0.01 M EDTA, pH=8). After resuspension of the pellet, 200 µl of lysozyme (10 mg ml^−1^) were added and incubated for 60 min at 37 °C. In brief, 40 µl of 20% SDS and 8 µl of proteinase K (20 mg ml^−1^) were added, and the samples were vortexed and incubated for 60 min at 60 °C. In brief, 300 µl of 6 M NaCl was added and vortexed repeatedly for 15 s and centrifuged at 4 °C for 30 min at 11,000 ***g***. The supernatant was collected and centrifuged for 10 min at the same conditions; the supernatant was collected, and 300 µl of cold isopropanol was added to precipitate nucleic acids at −20 °C for 60 min and centrifuged at 4 °C (11,000 ***g***) for 20 min. Importantly, 70% of ethanol was used to wash the DNA pellet; the DNA was resuspended in 100 µl of MilliQ sterile water. Then, 3 µl RNAase (1 mg ml^−1^) was added and incubated for 60 min at 37 °C. The quality of gDNA was analysed by agarose electrophoresis at 1% and quantified by spectrometry (NanoDrop1000, Thermo Scientific).

Taxonomic identification based on the 16S rRNA sequence has been widely used elsewhere, which is based on the amplification of the 16S ribosomal subunit gene (~1.5 Kbp); partially targeted regions have been used depending on the sample availability and sequencing technology facility. The V1-V3 region has been used for the identification of environmental and infant human gut microbiomes, but it’s been reported to have limited coverage on some genera or species [24,25]. Even with the limitations, this region is considered sufficient and accurate for the identification of most genera and species. We used V1-V3 universal oligonucleotides 27F/534R (5′-TTGGAGAGTTTGATCMTGGCTC-3′ and 5′-GTATTACCGCGGCTGCTG-3′) to amplify the first 500 bp region, considering CLSI MM-18 criteria [26] to get an approach to the identification of the samples. PCR conditions were 1 cycle of 94 °C for 3 min and 40 cycles of 94 °C for 45 s and 64 °C for 30 s annealing, 72 °C for 50 s elongation and final extension of 72 °C for 5 min using Dream Taq polymerase (Thermo Scientific) [27]. The products were purified using the GeneJET gel extraction kit (Thermo Scientific) following the manufacturer’s instructions; the samples were sequenced using the Sanger method at LabSerGen-LANGEBIO, Irapuato, Mexico. Repeated readings were performed on the samples; to minimize the error, the sequences were analysed (based on quality score or Phred value); low-quality bases at the edge were trimmed; and forward and reverse reads were combined using the software Geneious Prime v2024.0 (https://www.geneious.com). Database analysis was based on GeneBank of the National Center for Biotechnology Information (https://www.geneious.com), and the highest identity, sequence coverage and expectation value were used to set genus and/or species identification.

## Results

**Fig. 1.**
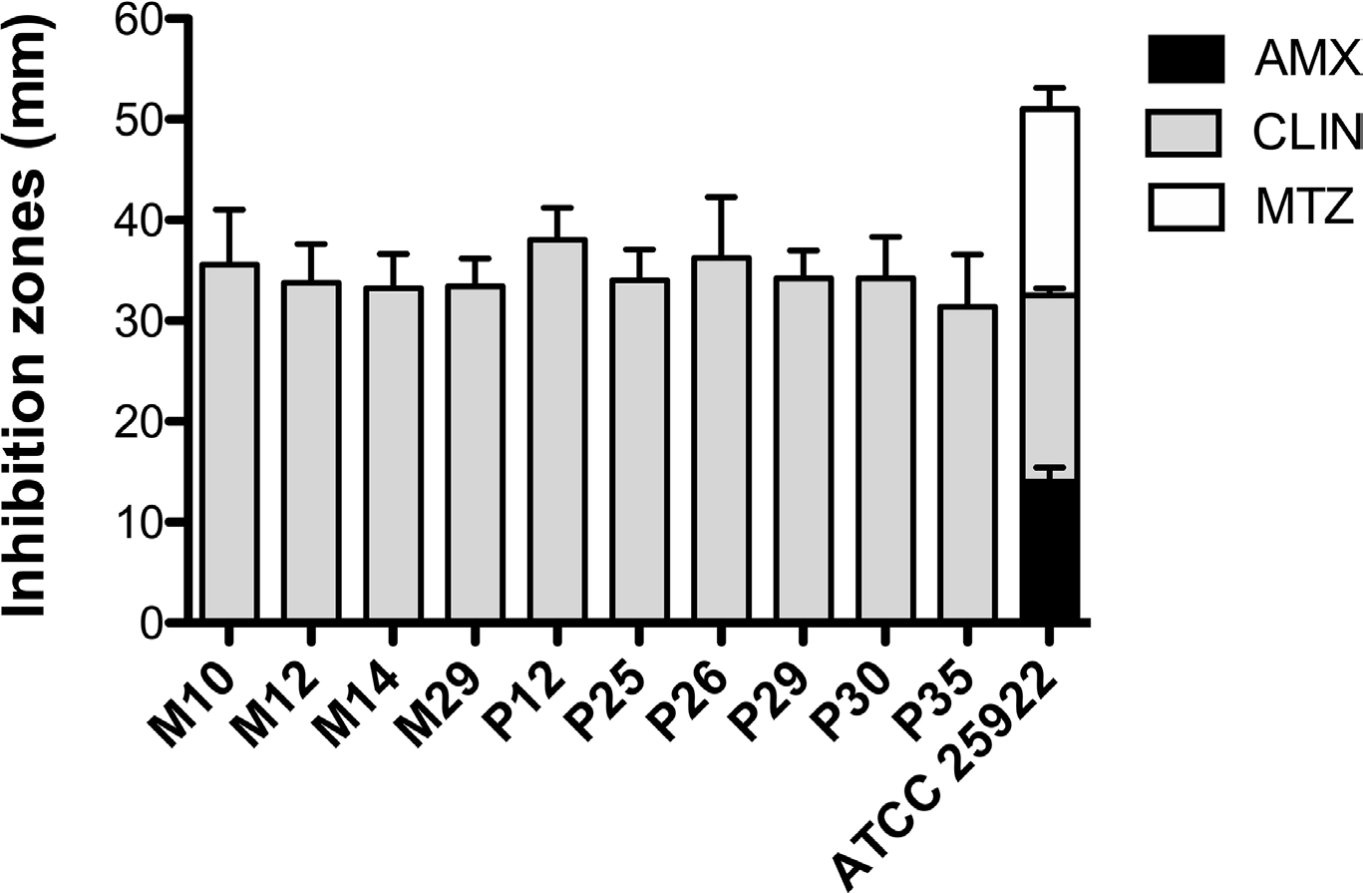
Bar graph showing antibiotic inhibition zones for peri-implant bacteria. Clinical isolates (n=10) mainly display sensitivity to clindamycin (CLIN) with zones of 31-38 mm, and resistance to amoxicillin (AMX) and metronidazole (MTZ). While *E*. *coli* ATCC 25922 used as control strain exhibits sensitivity to all antibiotics tested. Experiments were repeated five times; inhibition zones in millimetres (mm) are expressed as mean±sd. M=isolates from implants with peri-implant mucositis (*n*=4); *P*=isolates from implants with peri-implantitis (*n*=6).

**Fig. 2.**
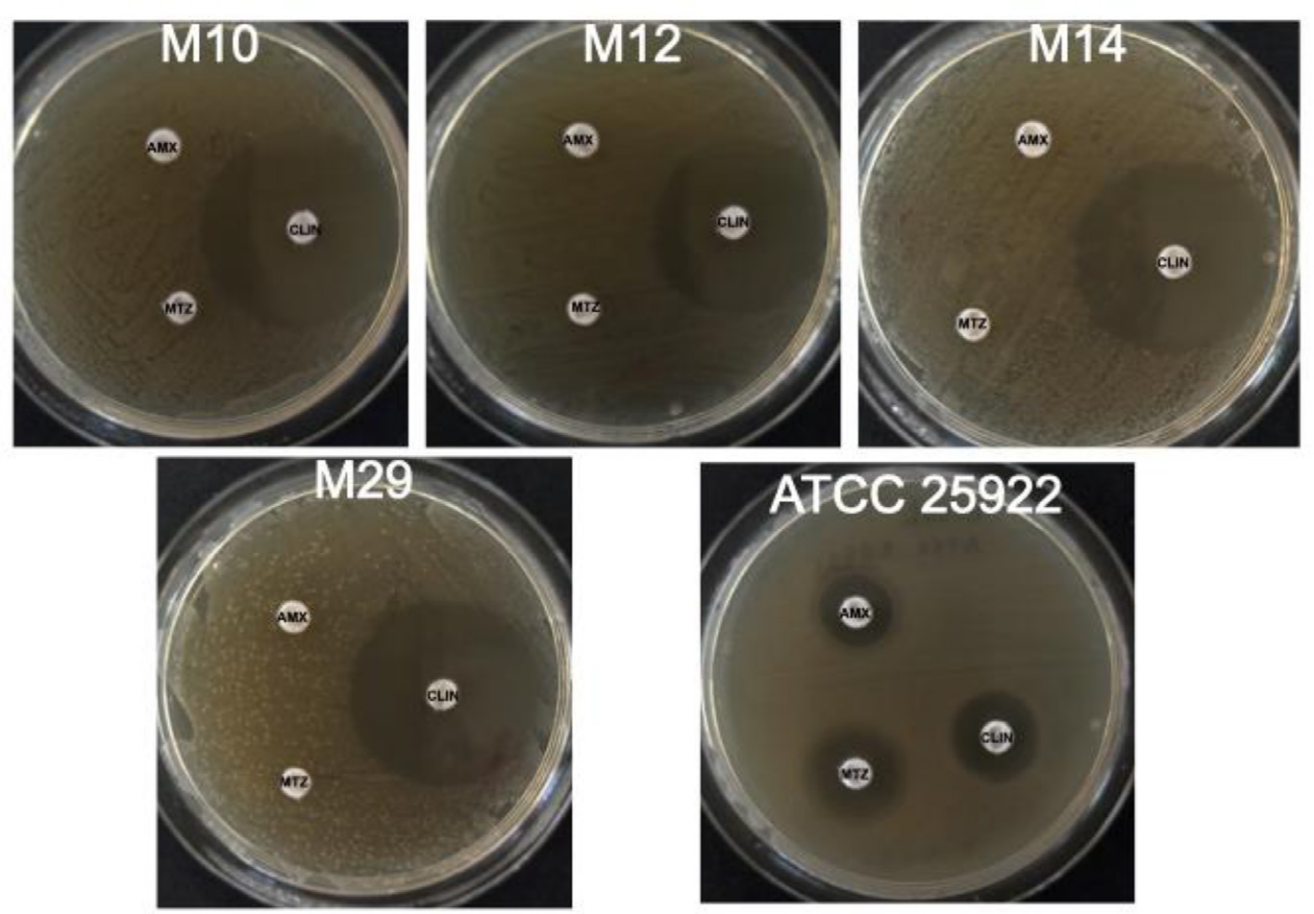
Disc diffusion susceptibility assay of bacteria isolated from implants with peri-implant infections. Representative assays of microorganisms recovered from implants with peri-implant mucositis. Discs were impregnated with 10 µg of amoxicillin (AMX), 30 µg of clindamycin (CLIN) and 50 µg of metronidazole (MTZ). *E. coli* ATCC 25922 was used as control strain. Experiments were repeated five times, and representative pictures are shown for each strain.

To analyse amoxicillin, clindamycin and metronidazole resistance of bacteria associated with peri-implant infections, ten biofilms were collected from the deeper regions of cylindrical screw-thread implants with infections. In these regions, known as the apex, low oxygen concentrations favour the proliferation of anaerobic micro-organisms. Bacterial cultivation revealed facultative anaerobes. The antimicrobial susceptibility profiles of ten peri-implant isolates are illustrated in Fig. 1. An antibiotic resistance to amoxicillin and metronidazole was observed in ten out of ten samples, representing a resistance of 100% of isolates (Table 1). Because isolates grew up to the edge of the discs when amoxicillin and metronidazole were tested, inhibition zones were reported as 0 mm (Table 1 and Figs2  3) [19]. In contrast, clindamycin mediated an inhibition of bacterial growth of isolates recovered from implants with peri-implant mucositis: M10 (35.6±5.4), M12 (33.8±3.8), M14 (33.2±3.4) and M29 (33.4±2.8) (Fig. 1 and Table 1). A similar antimicrobial resistance profile was found in bacteria isolated from implants with peri-implantitis: P12 (38.0±3.2), P25 (34.0±3.1), P26 (36.2±6.1), P29 (34.2±2.8), P30 (34.2±4.1) and P35 (31.4±5.2) (Fig. 1 and Table 1). *E. coli* ATCC 25922 showed a sensibility to amoxicillin (14.0±1.4), metronidazole (18.5±2.1) and clindamycin (18.5±0.7) (Table 1). Figs2  3 show representative images of disc diffusion susceptibility assays of ten peri-implant strains: M10, M12, M14, M29, P12, P25, P26, P29, P30 and P35.

**Table 1.** Antibiotic sensibility to amoxicillin, clindamycin and metronidazole in clinical isolates from peri-implant infections Antibiotic sensibility testing was evaluated in peri-implant bacteria isolated from explanted dental implants with peri-implant mucositis (*n*=4) or peri-implantitis (*n*=6). Amoxicillin, clindamycin and metronidazole were used at a concentration of 10, 30 and 50 μg, respectively. Experiments were repeated five times; inhibition zones in millimetres (mm) are expressed as mean±sd. *E. coli* ATCC 25922 was used as a negative control strain. r=resistant; s=sensitive.

Strain	Peri-implant disease	Zone diameter inhibition (mm) Amoxicillin	Zone diameter inhibition (mm) Clindamycin	Zone diameter inhibition (mm) Metronidazole
M10	Mucositis	0 (r)	35.6±5.4 (s)	0 (r)
M12	Mucositis	0 (r)	33.8±3.8 (s)	0 (r)
M14	Mucositis	0 (r)	33.2±3.4 (s)	0 (r)
M29	Mucositis	0 (r)	33.4±2.8 (s)	0 (r)
P12	Peri-implantitis	0 (r)	38.0±3.2 (s)	0 (r)
P25	Peri-implantitis	0 (r)	34.0±3.1 (s)	0 (r)
P26	Peri-implantitis	0 (r)	36.2±6.1 (s)	0 (r)
P29	Peri-implantitis	0 (r)	34.2±2.8 (s)	0 (r)
P30	Peri-implantitis	0 (r)	34.2±4.1 (s)	0 (r)
P35	Peri-implantitis	0 (r)	31.4±5.2 (s)	0 (r)
*E. coli* ATCC 25922		14±1.4 (s)	18.5±0.7 (s)	18.5±2.1 (s)

**Fig. 3.**
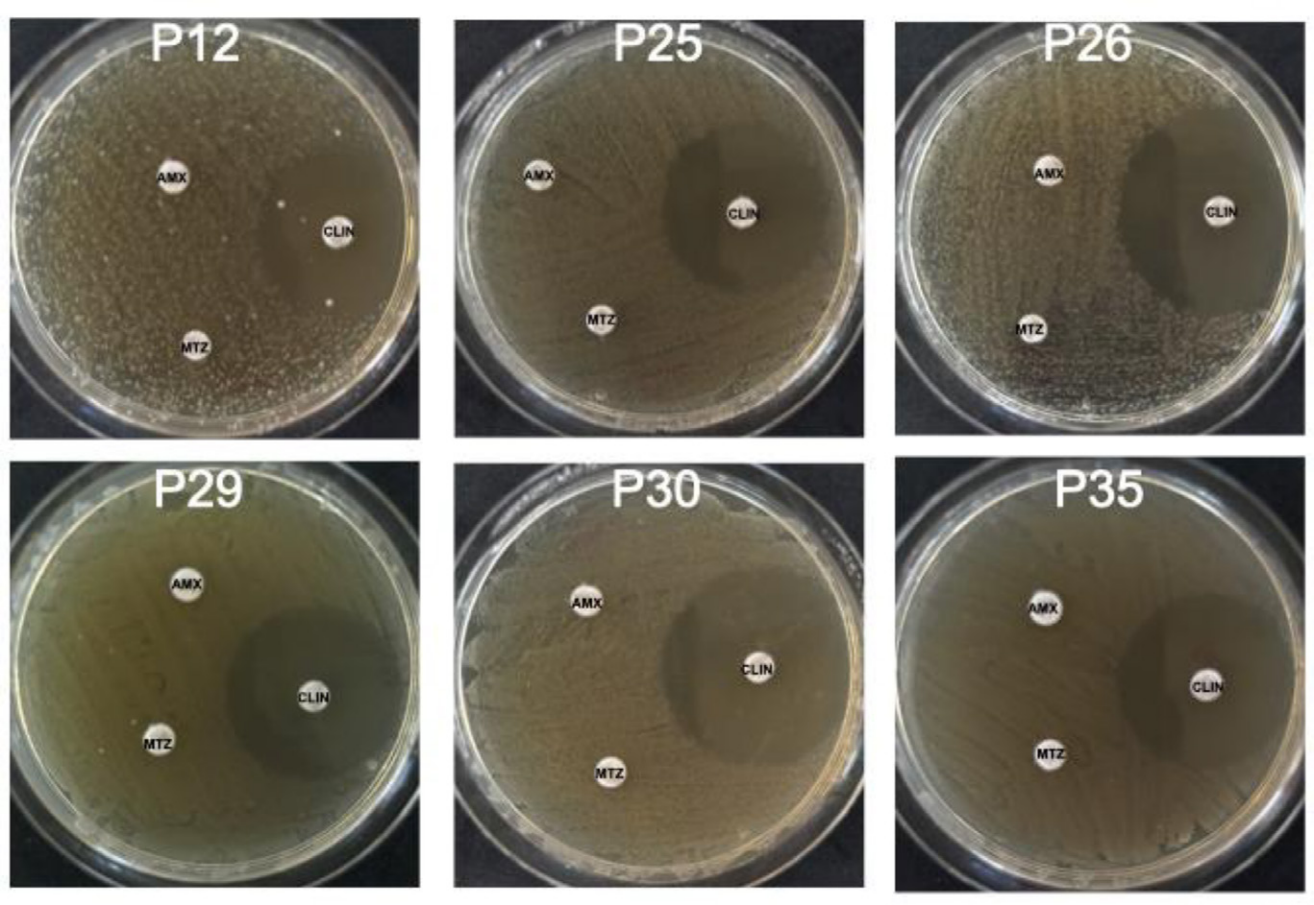
Disc diffusion susceptibility assay of bacteria isolated from implants with peri-implant infections. Representative assays of microorganisms recovered from implants with peri-implantitis. Discs were impregnated with 10 µg of amoxicillin (AMX), 30 µg of clindamycin (CLIN) and 50 µg of metronidazole (MTZ). Experiments were repeated five times, and representative pictures are shown for each strain.

To identify the bacterial species involved in peri-implant infections, strains M29 (from mucositis) and P30 (from peri-implantitis) were selected. Both isolates were cultured over multiple passages, and a single smooth-shaped colony was observed. Gram staining revealed Gram-positive cocci forming short chains in strains M29 and P30 (File S1, available in the online Supplementary Material). Additionally, both isolates were catalase- and oxidase-negative (data not shown). Sequencing of their 16S rRNA identified *Streptococcus salivarius* with 99.8% identity for the M29 strain and 100% identity for the P30 strain (data not shown).

Our findings reveal emerging resistance to amoxicillin and metronidazole in ten clinical isolates recovered from the apex of dental implants with peri-implant infections, yet bacterial susceptibility to clindamycin remains.

## Discussion

The objective of the present study was to evaluate the antibiotic resistance of bacteria found at subgingival plaque of dental implants with peri-implant infections. Amoxicillin, clindamycin and metronidazole were tested. In total, 100% of clinical isolates were resistant to amoxicillin and metronidazole, yet susceptibility to clindamycin remains (Fig. 1 and Table 1). Previous findings showed antimicrobial resistance in bacteria isolated from dental implants; van Winkelhoff and Wolf found resistance to metronidazole in *Aggregatibacter actinomycetemcomitans* isolated from an edentulous patient with peri-implantitis [28]. Karbach *et al.* analysed 138 isolates from implants; of them, 38 (27.6%) were resistant to antibiotics [29]. Rams *et al.* studied subperiosteal implants with peri-implantitis and observed resistance to doxycycline in one of three patients [30]. A follow-up study by the same group found that 71.7% of micro-organisms cultured from submucosal biofilms from patients with peri-implantitis exhibited antimicrobial resistance, with 6.7% of bacteria resistant to amoxicillin and metronidazole [20]. In 2016, Koukos *et al.* studied 20 implants with peri-implantitis and found that 15/20 (75%) of implants carried the *te*tQ gene and 8/20 (40%) of implants carried the *tet*M gene (both of which confer resistance to tetracycline) and 1/20 (5%) of implants carried the *bla*_TEM_ gene, which is associated with resistance to *β*-lactam antibiotics [31]. These findings suggest that certain bacteria isolated from implants with peri-implantitis were resistant to metronidazole, doxycycline, amoxicillin and tetracycline.

Our results are consistent with the antibiotic resistance of bacteria to amoxicillin and metronidazole [20,28], but antibiotic resistance to doxycycline and tetracycline remains to be tested. Amoxicillin, a *β*-lactam antibiotic, is the first-choice antibiotic for treating peri-implant infections. For patients allergic to amoxicillin, clindamycin, a lincosamide antibiotic targeting *β*-lactam-resistant bacteria, is recommended. Combination of amoxicillin and metronidazole is also used to improve the surgical outcome of peri-implantitis [32].

In contrast with the study of Rams *et al.*, who found clindamycin resistance in bacteria isolated from submucosal biofilm [20], we did not find resistance to clindamycin (Table 1). While submucosal biofilm recovered micro-organism from tissue, subgingival biofilm recovered bacteria from dental implants; such differences in biofilms could have an effect on antimicrobial resistance profile.

To characterize the micro-organisms that colonize dental implants, we sequenced their 16S rRNA and conducted a Gram stain (File S1) and catalase and oxidase test (data not shown). One isolate from implants with peri-implant mucositis, M29, and one isolate from implants with peri-implantitis, P30, were selected. Both isolates were resistant to amoxicillin and metronidazole (Table 1). Both isolates were identified as *S. salivarius*, which is consistent with the presence of this micro-organism in dental implants with peri-implantitis [33].

## Conclusion

Our findings reveal emerging resistance to amoxicillin and metronidazole in ten clinical isolates from implants with peri-implant infections, yet bacterial susceptibility to clindamycin remains. Monitoring antibiotic resistance profiles could help limit the spread of bacterial resistance. In cases where resistance to amoxicillin and metronidazole is detected, clindamycin should be considered for use in dental practice.

## Limitations

With the exception of the patient from whom M29 and P30 samples were collected, clinical and demographic information was not obtained for other patients with peri-implant diseases in this study.

## Supplementary material

10.1099/acmi.0.000946.v3 Uncited Fig. S1.

## Abbreviations

BOP: bleeding on probing
CLSI: Clinical Laboratory Standards Institute
PD: probing depth
