# Supplementary material for: Amoxicillin and metronidazole resistance of bacteria isolated from dental implants with peri-implant diseases: a pilot cross-sectional study
Source: Access Microbiol. 2026 Feb 11;8(2):000946.v3. doi: 10.1099/acmi.0.000946.v3 (PMC12909538; doi:10.1099/acmi.0.000946.v3)
Supplement: Uncited Fig. S1. [file acmi-8-00946-s001.pdf]

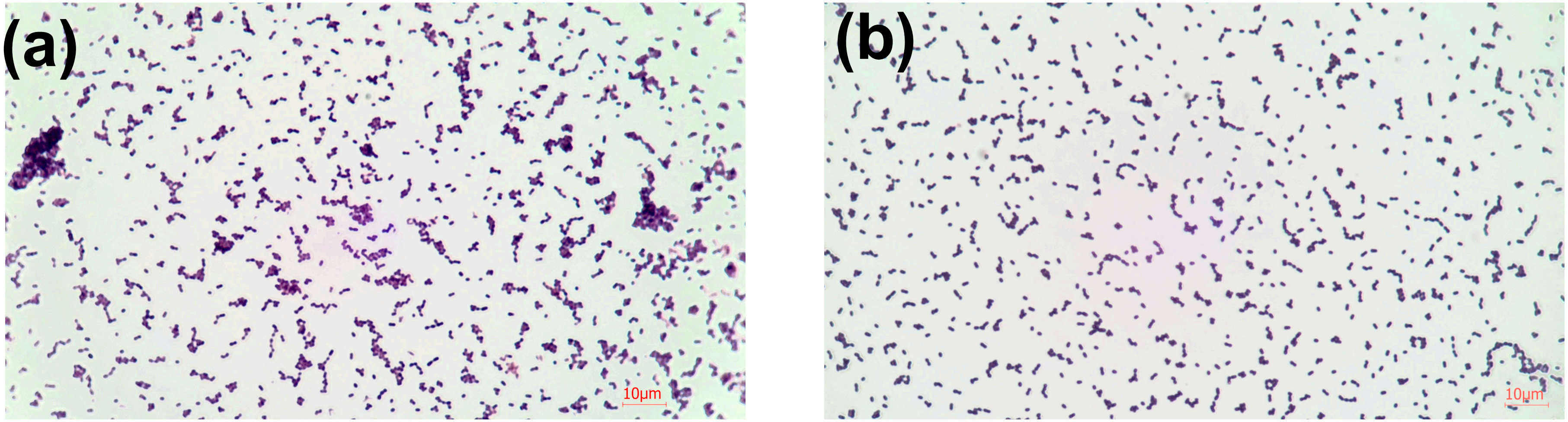

Supplemental 1. Gram staining of bacteria isolated from dental implants with peri-implant mucositis (M29) and peri-implantitis (P30). Gram-positive cocci forming short chains were observed in the strains M29 (a) an P30 (b).
